# Supplementary material for: Molecular basis for the specific and multivariant recognitions of RNA substrates by human hnRNP A2/B1
Source: Nat Commun. 2018 Jan 29;9:420. doi: 10.1038/s41467-017-02770-z (PMC5789076; doi:10.1038/s41467-017-02770-z)
Supplement: Supplementary file 1 — Supplementary Information [file 41467_2017_2770_MOESM1_ESM.pdf]

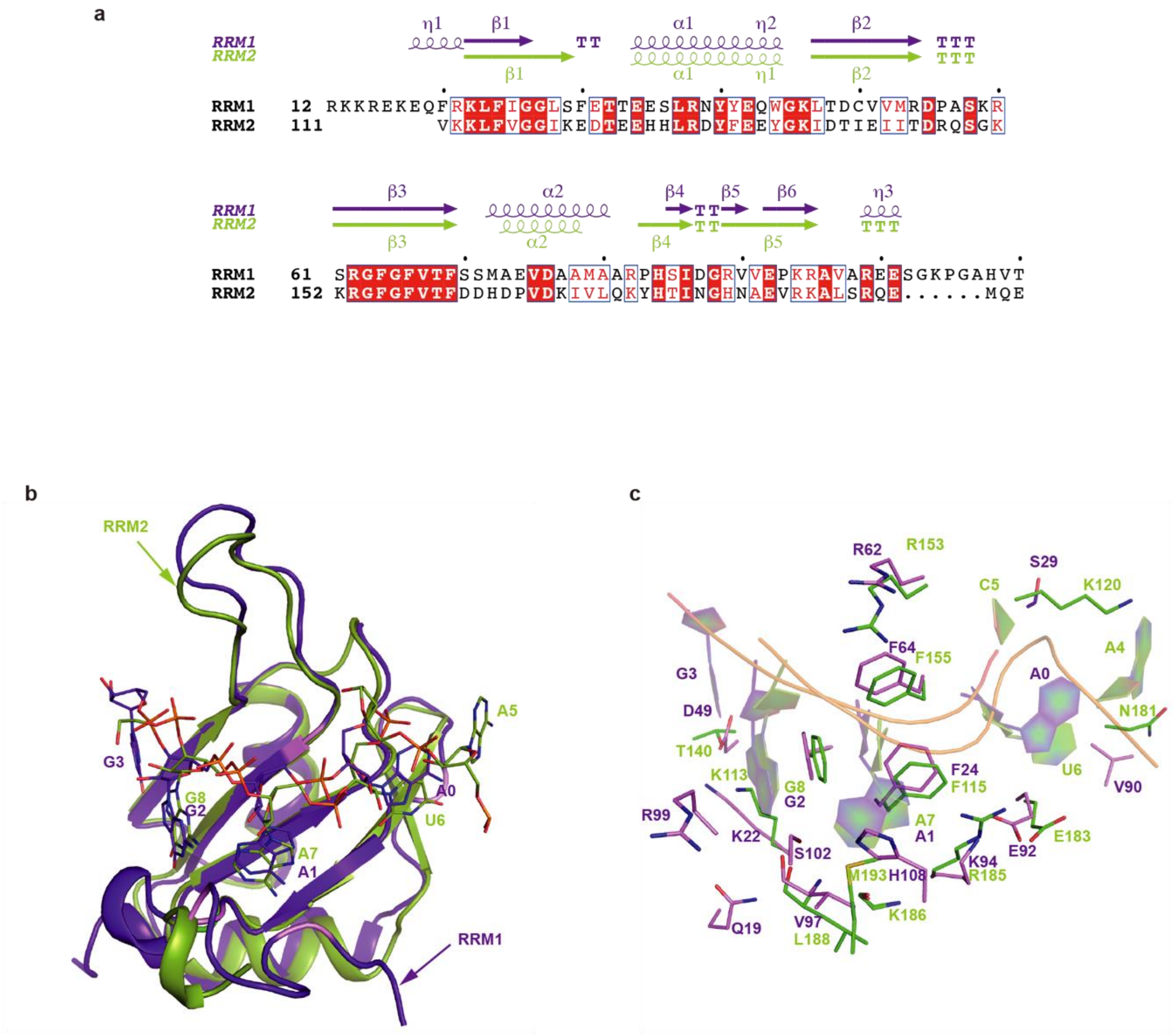

Supplementary Figure 1. Superimposed of RRM1 and RRM2 in 10mer RNA complex

(a) Sequence alignment between RRM1 and RRM2, the secondary structures are colored in purple-blue and green, respectively above the sequences. (b) Superposition of RRM1(12-110)-AAGG and RRM2(111-195)-ACUAGC.(c) Superposition of residues participated in RNA recognition in RRM1 and RRM2. The RNA chains are shown by cartoon.

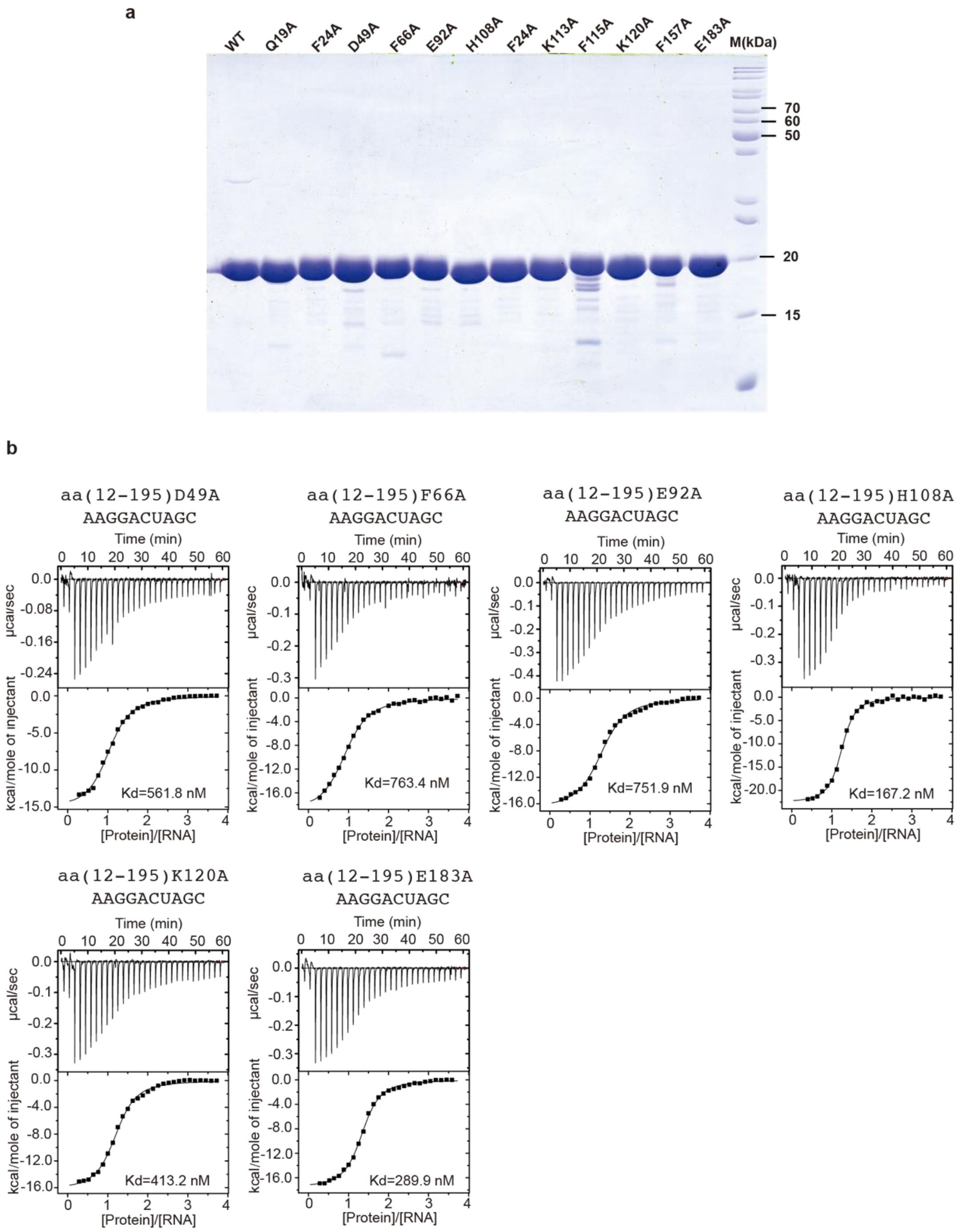

Supplementary Figure 2. Mutagenesis study of RRMs by ITC experiments

(a) SDS-PAGE of wildtype and protein mutants. (b) ITC results of hnRNP A2/B1 mutants with 10mer RNA.

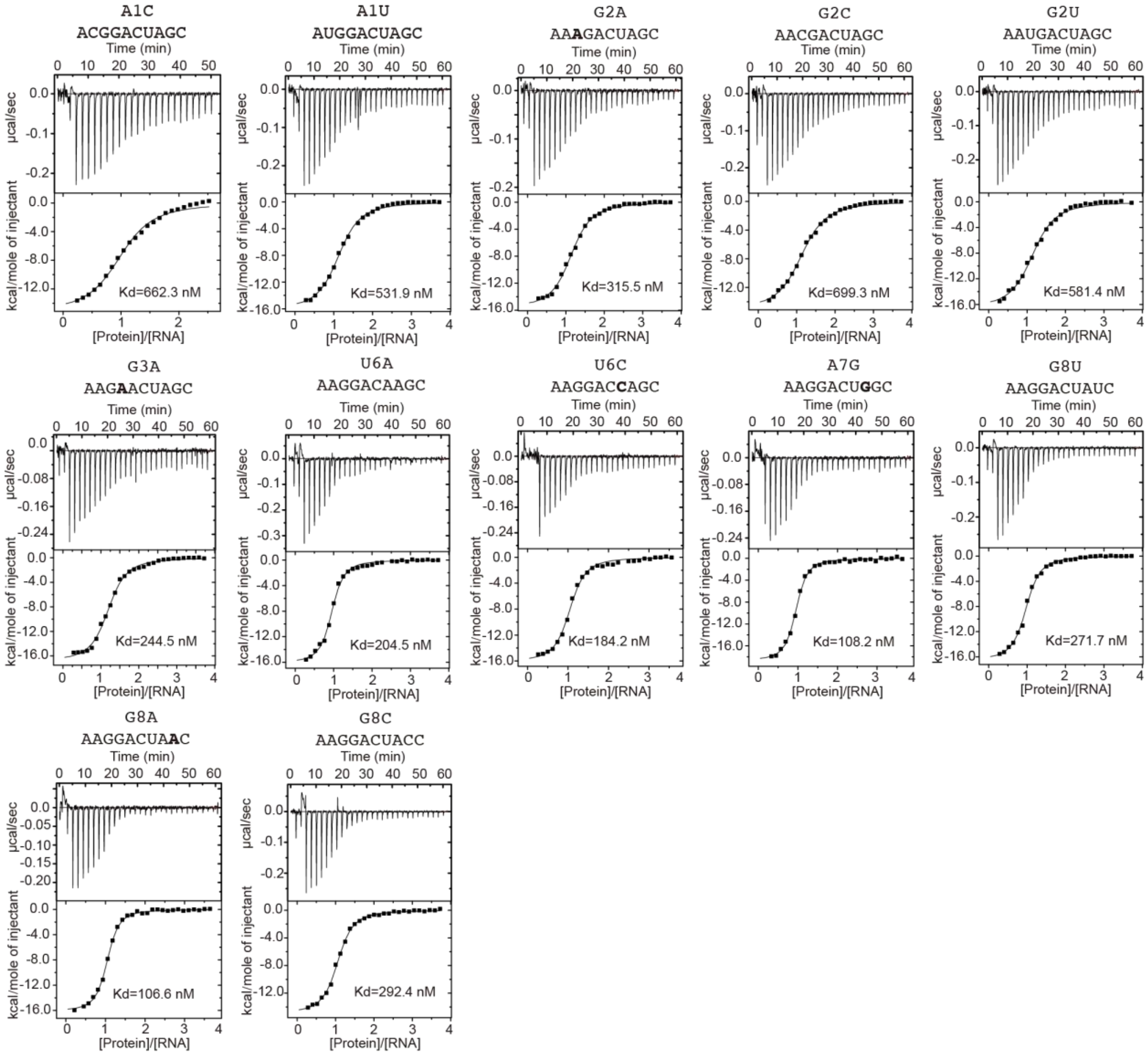

Supplementary Figure 3. Mutagenesis study of RNA by ITC experiments.

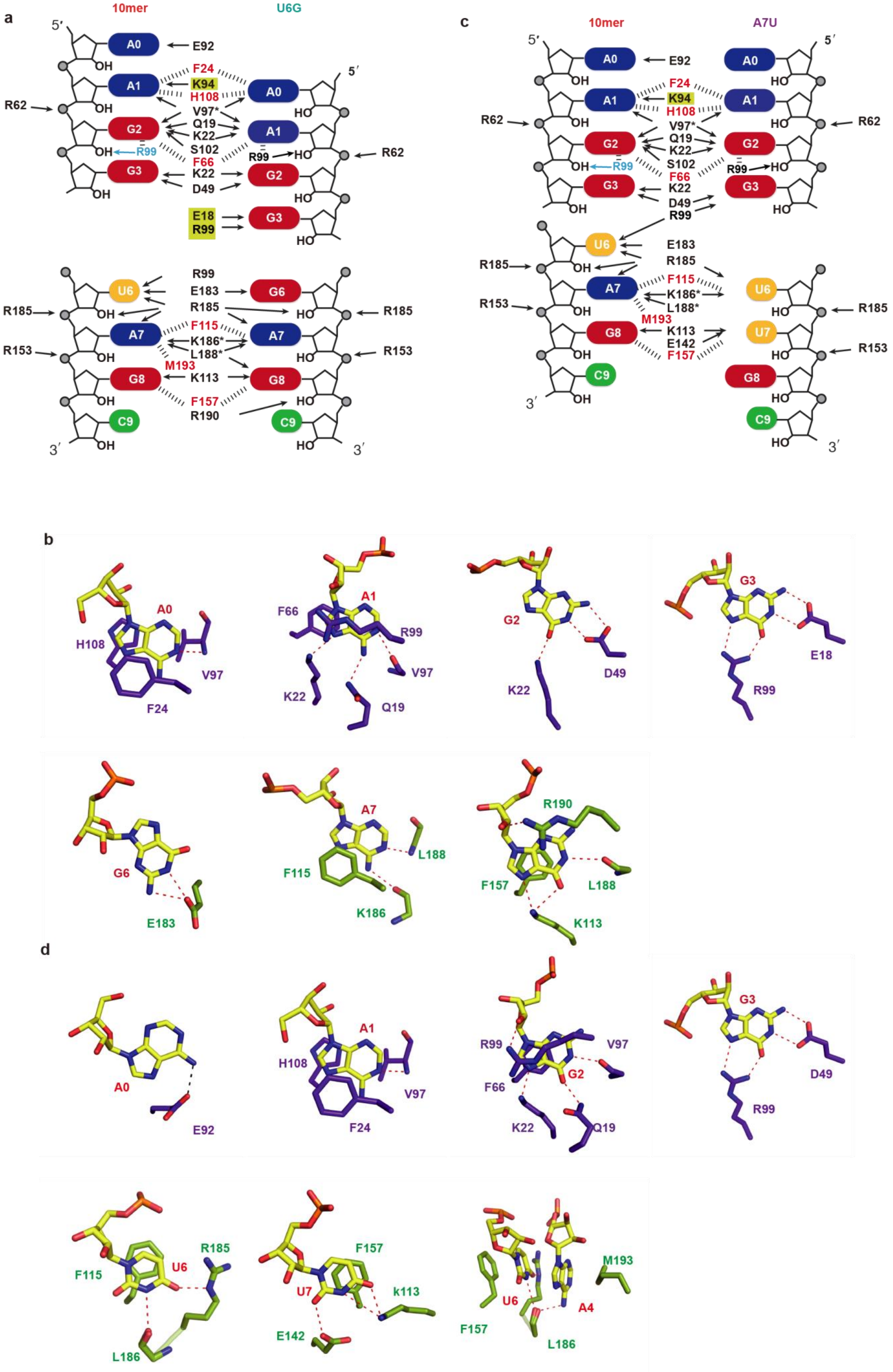

a
10mer
U6G
5′
A0
E92
OH
A1
F24
K94
H108
V97*
Q19
K22
S102
F66
R62
G2
R99
G3
D49
E18
A7
R185
R153
U6
E183
G6
F115
K186*
L188*
M193
G8
K113
F157
R190
C9
HO
3′
c
A7U
U7
E142
b
H108
V97
F24
K22
Q19
D49
d
A4
L186
k113

Supplementary Figure 4. Schematic representations of RRMs interactions with RNA mutants. (a) Close-up view showing the specific RNA recognition in U6G complex. (b) Comparison of detailed interactions between 10mer complex and U6G complex. (c) Close-up view showing the specific RNA recognition in A7U complex. (d) Comparison of detailed interactions between 10mer complex and A7U complex.

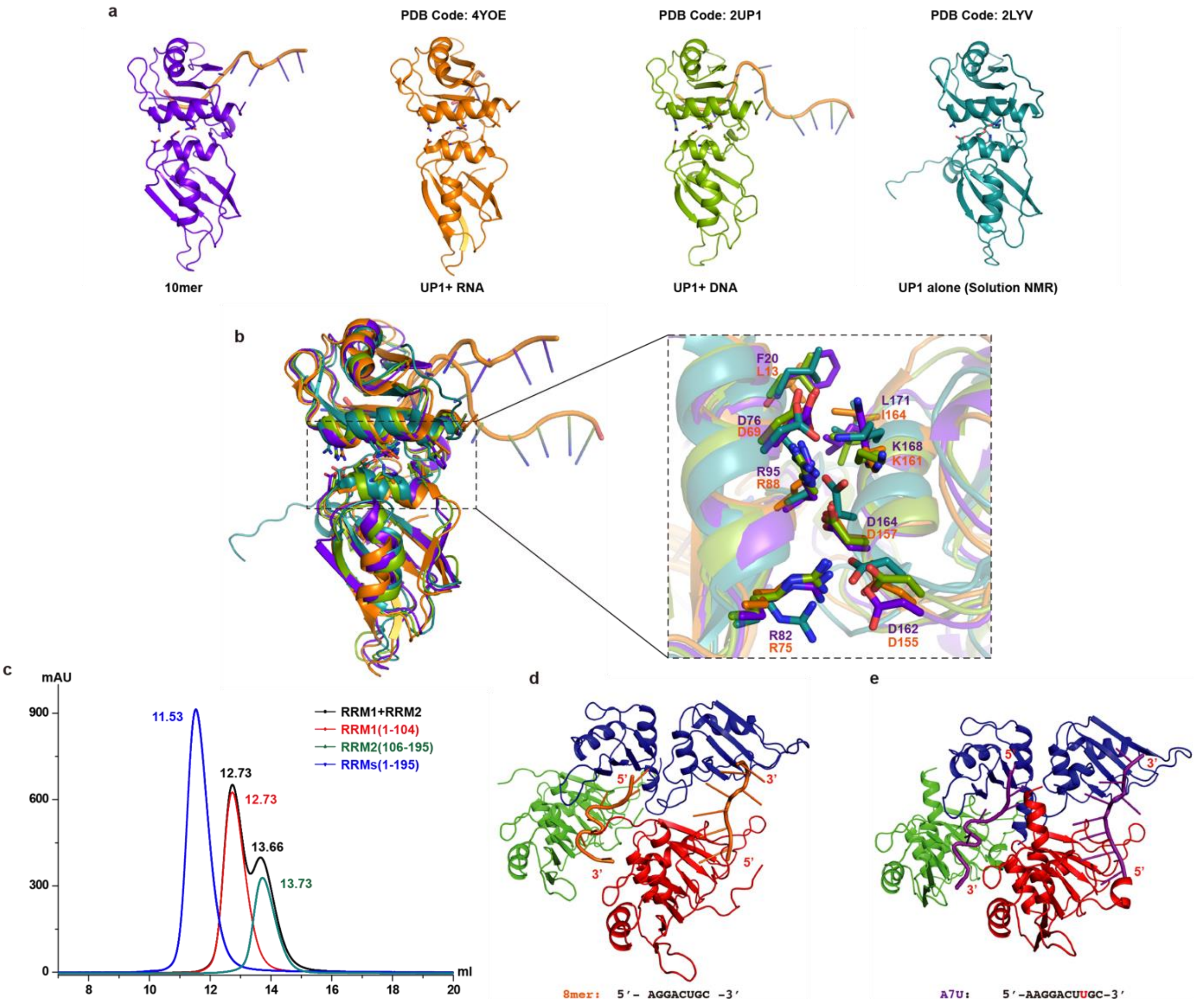

Supplementary Figure 5. Crystal packing interactions. (a) Structures of hnRNP A2/B1with 10mer complex and hnRNP A1-RRMs (UP1) in different structural status. The UP1 in complex with RNA (PDB code: 4YOE) is colored in orange, the UP1 in complex with telomere DNA (PDB code: 2UP1) and solution structure of UP1 (PDB code: 2LYV) are colored in green and cyan, respectively. (b) Superposition of the structures listing in Supplementary Figure 5a reveals the conserve interactions between RRM1 and RRM2. The residues account for the interactions of inter-domain are shown as stick colored as above. (c) Gel-filtration binding assay for RRM1 and RRM2. Black curve: RRM1+RRM2; Red curve: RRM1; Green curve: RRM2; Blue curve: RRMs(1-195). (d) Crystal packing interactions in 8mer. (e) Crystal packing interactions in A7U complex.

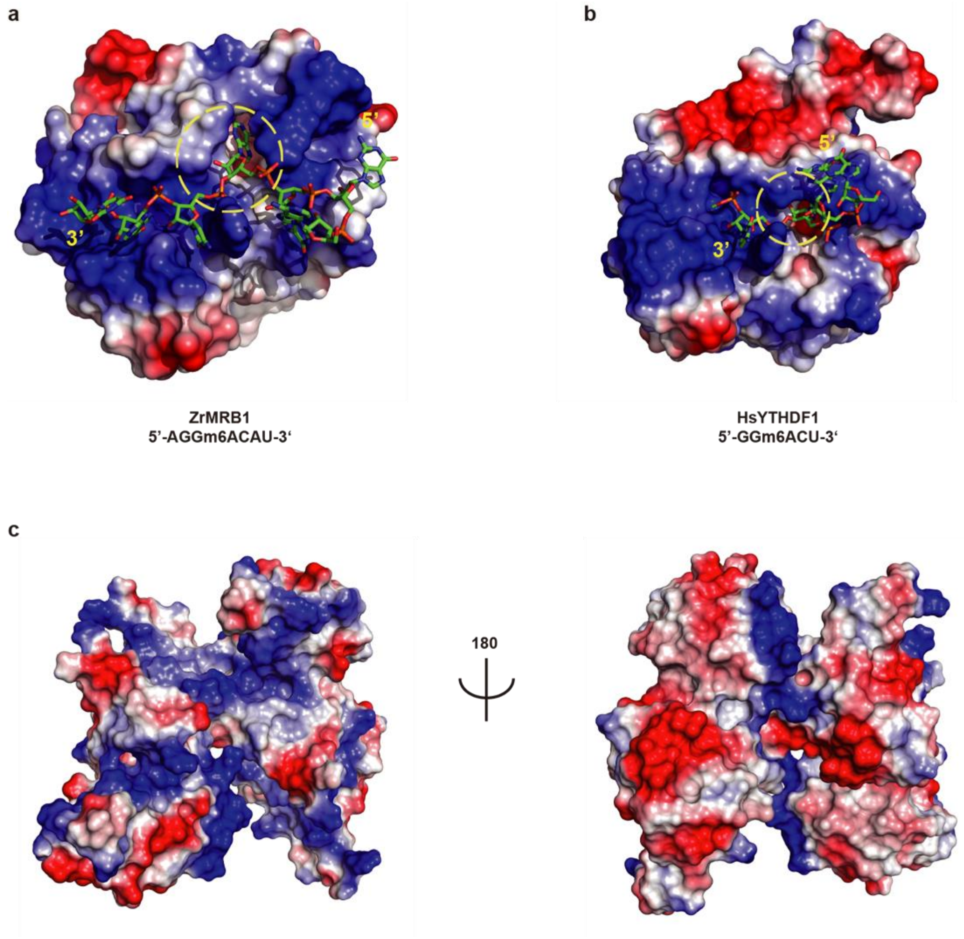

Supplementary Figure 6. Surface charge comparison of *Zr*MRB1, *Hs*YTHDF1 and hnRNP A2/B1. (a) Surface representation of *Zr*MRB1 in complex with RNA containing m^{6}A modification. The aromatic cage is circled with yellow dashline, RNA is shown by stick colored in green. (b) *Hs*YTHDF1 in complex with RNA containing m^{6}A modification, RNA is shown by stick. (c) Surface representation of hnRNP A2/B1 RRMs in complex with 10mer RNA.

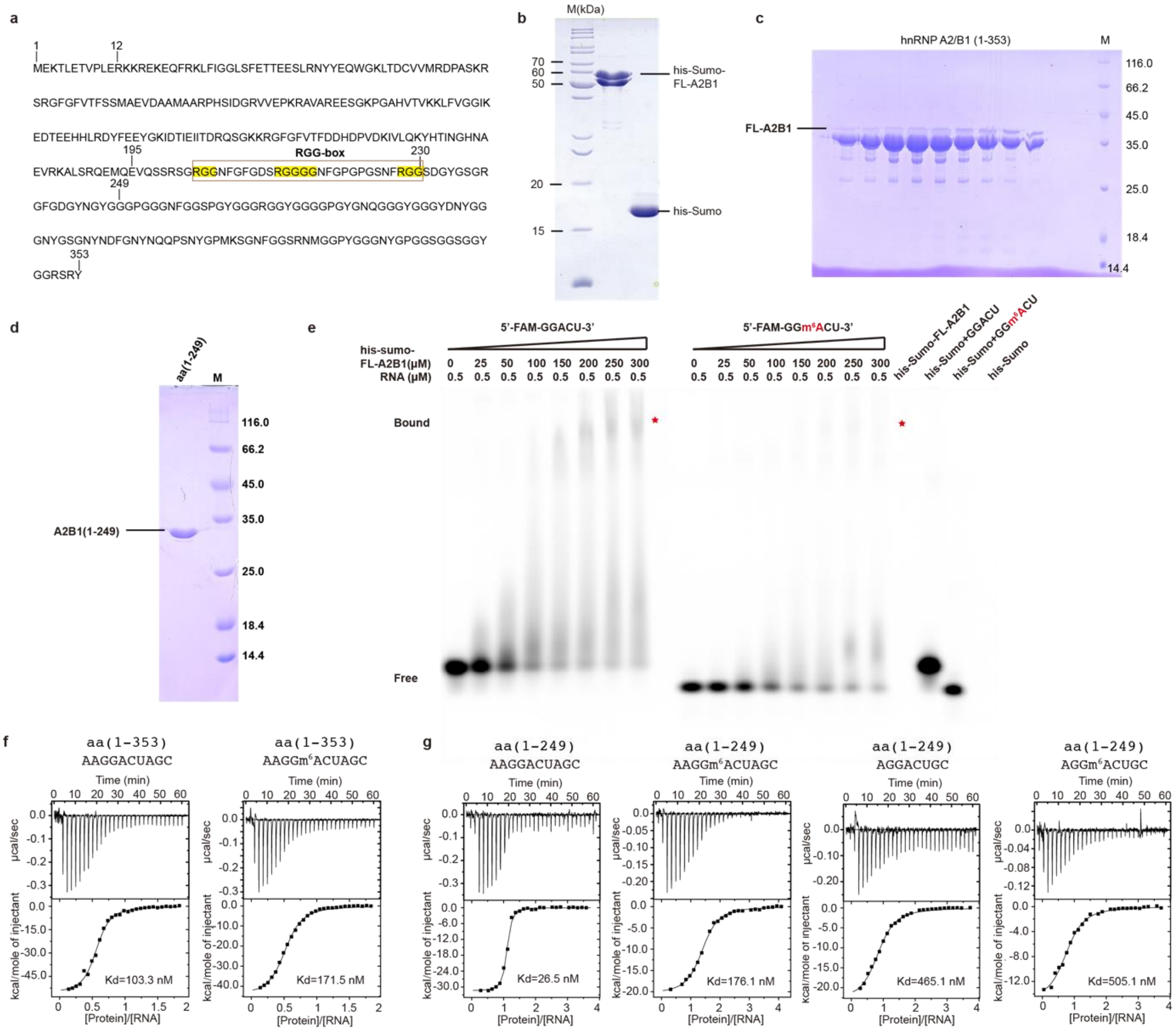

Supplementary Figure 7. Full-length hnRNP A2/B1 does not specifically recognize m^{6}A-modified RNA. (a) Sequence of full-length hnRNP A2/B1 with RGG-box (203-230) colored in yellow enclosed with rectangle. (b) SDS-PAGE of full-length hnRNP A2/B1 with his-sumo tag. (c) SDS-PAGE of full-length hnRNP A2/B1 without tag. (d) SDS-PAGE of hnRNP A2/B1(aa 1-249) containing the RGG-box. (e) EMSA result of full-length hnRNP A2/B1 and 5'-FAM-labeled RNA substrates with or without m^{6}A modification. (f) ITC results of full-length hnRNP A2/B1 with various RNA substrates. (g) ITC results of hnRNP A2/B1(aa 1-249) with various RNA substrates.

Supplementary Table 1. Data collection and refinement statistics.

	aa (12-195) and 8mer AGGACUGC	aa (12-195) and 10mer AAGGACUAGC	aa (12-195) and A1G AGGGACUAGC	aa (12-195) and U6G AAGGACGAGC	aa (12-195) and G7U AAGGACUUGC
**Data collection**					
Space group	$C2_1$	$C2_1$	$P4_3$	$P2_1$	$C2_1$
Cell dimensions					
a , *b*, *c* (Å)	122.623, 46.38, 37.516	109.604, 38.959,70.442	62.591, 62.591, 53.043	49.351, 38.301, 107.931	132.810, 46.228, 33.994
α,β γ, (°)	90, 96.02, 90	90, 116.26, 90	90, 90, 90	90, 92.43, 90	90, 102.22, 90
Wavelength(Å)	0.97776	0.97776	0.97776	0.97776	0.97776
Resolution (Å)	28.81-2.60(2.64-2.6)	63.17-1.85 (1.917-1.851)	30.00-2.40(2.44-2.40)	30.00-2.15(2.23-2.15)	30-2.03(2.082-2.030)
R_{merge}	0.119	0.057	0.104	0.137	0.131
$I/\sigma I$	7.9 (1.5)	18.4 (2.1)	32.75(2.33)	11.0(2.25)	15.81(2.4)
Completeness (%)	93.4 (82.0)	92.43(55.36)	100.0(100)	96.8(87.9)	97.2(94.0)
Redundancy	2.5	2.9	13.4	4.7	4.9
**Refinement**					
Resolution (Å)	2.6	1.85	2.4	2.15	2.03
No. reflections	15240 (461)	21311(1255)	8124(392)	21583(1917)	14087(1172)
R_{work} / R_{free}	0.1916/0.2362	0.1839/0.2227	0.22436/.27434	0.20255/0.25772	0.20145/0.25628
No.atoms	1652	1796	1637	3399	1734
Protein	1493	1470	1433	2903	1522
RNA	149	193	198	436	169
Water	10	134	6	60	33
Average *B*-factor (Å)	45.5	23.57	68.279	29.476	42.795
**R.m.s. deviations**					
Bond lengths (Å)	0.012	1.018	0.012	0.013	0.016
Bond angles (°)	1.7	1.95	1.581	1.757	1.831
**Ramachandran plot**					
Favored/allowed/ disallowed(%)	96.00/4.00/0.00	99.00/1.00/0.00	94.51/2.44/3.05	98.21/0.9/0.9	98.27/0.58/1.16

Supplementary Table 2. Summary of ITC results

RNA substrates	Protein	Kd	n
8mer: AGGACUGC	hnRNP A2/B1 (12-195)	276.2 ± 27.5 nM	0.826
10mer: AAGGACUAGC	hnRNP A2/B1 (12-195)	114.7 ± 11.5 nM	1.09
10mer-m^6A: AAGG(m^6A)CUAGC	hnRNP A2/B1 (12-195)	1.32 ± 0.143 μM	0.948
8mer-m^6A: AGG(m^6A)CUGC	hnRNP A2/B1 (12-195)	460.8 ± 47.4 nM	0.963
A1G: AGGGACUAGC	hnRNP A2/B1 (12-195)	534.8 ± 40.3 nM	1.01
A1C: ACGGACUAGC	hnRNP A2/B1 (12-195)	662.3 ± 54.3 nM	1.02
A1U: AUGGACUAGC	hnRNP A2/B1 (12-195)	531.9 ± 66.7 nM	1.14
G2A: AAAGACUAGC	hnRNP A2/B1 (12-195)	315.5 ± 37.0 nM	1.14
G2C: AACGACUAGC	hnRNP A2/B1 (12-195)	699.3 ± 88.5 nM	1.17
G2U: AAUGACUAGC	hnRNP A2/B1 (12-195)	581.4 ± 58.1 nM	1.17
G3A: AAGAACUAGC	hnRNP A2/B1 (12-195)	244.5 ± 25.3 nM	1.19
G3C: AAGCACUAGC	hnRNP A2/B1 (12-195)	952.4 ± 1.14 nM	1.1
U6C: AAGGACCAGC	hnRNP A2/B1 (12-195)	184.2 ± 16.3 nM	1.03
U6A: AAGGACAAGC	hnRNP A2/B1 (12-195)	204.5 ± 19.9 nM	0.924
U6G: AAGGACGAGC	hnRNP A2/B1 (12-195)	238.1 ± 42.7 nM	1.08
A7G: AAGGACUGGC	hnRNP A2/B1 (12-195)	108.2 ± 11.5 nM	0.919
A7U: AAGGACUUGC	hnRNP A2/B1 (12-195)	219.8 ± 21.6 nM	0.904
G8A: AAGGACUAAC	hnRNP A2/B1 (12-195)	106.6 ± 16.2 nM	1.01
G8C: AAGGACUACC	hnRNP A2/B1 (12-195)	292.4 ± 44.2 nM	1.04
G8U: AAGGACUAUC	hnRNP A2/B1 (12-195)	271.7 ± 30.7 nM	0.954
10mer:AAGGACUAGC	hnRNP A2/B1 (12-195)Q19A	440.5 ± 42.0 nM	1.19
10mer:AAGGACUAGC	hnRNP A2/B1 (12-195)F24A	456.6 ± 35.5 nM	1.03
10mer:AAGGACUAGC	hnRNP A2/B1 (12-195)D49A	561.8 ± 69.4 nM	1.04
10mer:AAGGACUAGC	hnRNP A2/B1 (12-195)F66A	763.4 ± 10.2 nM	0.952
10mer:AAGGACUAGC	hnRNP A2/B1 (12-195)E92A	751.9 ± 85.5 nM	1.3
10mer:AAGGACUAGC	hnRNP A2/B1 (12-195)H108A	167.2 ± 19.2 nM	1.21
10mer:AAGGACUAGC	hnRNP A2/B1 (12-195)K113A	813.0 ± 1.17 nM	1.27
10mer:AAGGACUAGC	hnRNP A2/B1 (12-195)F115A	781.3 ± 87.7 nM	1.31
10mer:AAGGACUAGC	hnRNP A2/B1 (12-195)K120A	413.2 ± 50.5 nM	1.2
10mer:AAGGACUAGC	hnRNP A2/B1 (12-195)F157A	534.8 ± 97.1 nM	1.39
10mer:AAGGACUAGC	hnRNP A2/B1 (12-195)E183A	289.9 ± 47.8 nM	1.32
10mer: AAGGACUAGC	hnRNP A2/B1 (1-353)	103.3 ± 8.93 nM	0.555
10mer-m^6A: AAGG(m^6A)CUAGC	hnRNP A2/B1 (1-353)	171.5 ± 25.6 nM	0.523
10mer: AAGGACUAGC	hnRNP A2/B1 (1-249)	26.5 ± 1.8 nM	1.05
10mer-m^6A: AAGG(m^6A)CUAGC	hnRNP A2/B1 (1-249)	176.1 ± 25.3 nM	1.19
8mer: AGGACUGC	hnRNP A2/B1 (1-249)	465.1 ± 53.2 nM	0.866
8mer-m^6A: AGG(m^6A)CUGC	hnRNP A2/B1 (1-249)	505.1 ± 44.2 nM	0.795

Supplementary Table 3. Potential targets for hnRNP A2/B1.

A2RE: 5’-GCCAAGGAGCCAGAGAGCAUG-3’	(JBC, 1999) [1]
A2RE11: 5’-GCCAAGGAGCC-3’	(JBC, 1999)[1]
CLIP-seq: 5’-GGUAGUAG-3’,	(Cell Reports, 2012) [2]
CLIP-seq: 5’-AGG(A/U)U(A/C/U)G(A/G)	
5’-UAASUUAU-3’	(Gene & Dev. 2016) [3]
5’-UAG(A/G)-3’	(Neuron, 2016) [4]
HOTAIR: Purine-rich	(RNA, 2016) [5]
5’-UAGGG-3’	(Nature, 2013) [6]
5’-GG(m6A)CU-3’	(Cell, 2015) [7]
5’-GAGG-3’	(Nat. Comms. 2013) [8]

Supplementary References

1. Munro, T.P. et al. Mutational analysis of a heterogeneous nuclear ribonucleoprotein A2 response element for RNA trafficking. *J Biol Chem* **274**, 34389-95 (1999).
2. Huelga, S.C. et al. Integrative genome-wide analysis reveals cooperative regulation of alternative splicing by hnRNP proteins. *Cell Rep* **1**, 167-78 (2012).
3. Geissler, R. et al. A widespread sequence-specific mRNA decay pathway mediated by hnRNPs A1 and A2/B1. *Genes Dev* **30**, 1070-85 (2016).
4. Hutten, S. & Dormann, D. hnRNPA2/B1 Function in Neurodegeneration: It's a Gain, Not a Loss. *Neuron* **92**, 672-674 (2016).
5. Meredith, E.K., Balas, M.M., Sindy, K., Haislop, K. & Johnson, A.M. An RNA matchmaker protein regulates the activity of the long noncoding RNA HOTAIR. *RNA* **22**, 995-1010 (2016).
6. Ray, D. et al. A compendium of RNA-binding motifs for decoding gene regulation. *Nature* **499**, 172-7 (2013).
7. Alarcon, C.R. et al. HNRNPA2B1 Is a Mediator of m(6)A-Dependent Nuclear RNA Processing Events. *Cell* **162**, 1299-308 (2015).
8. Villarroya-Beltri, C. et al. Sumoylated hnRNPA2B1 controls the sorting of miRNAs into exosomes through binding to specific motifs. *Nat Commun* **4**, 2980 (2013).
